# The machine learning methods to analyze the using strategy of antiplatelet drugs in ischaemic stroke patients with gastrointestinal haemorrhage

**DOI:** 10.1186/s12883-023-03422-0

**Published:** 2023-10-13

**Authors:** Chaohua Cui, Changhong Li, Min Hou, Ping Wang, Zhonghua Huang

**Affiliations:** 1 Department of Rehabilitation, Affiliated Hospital of Youjiang Medical University for Nationalities, zhongshaner road, youjiang District, Baise City, Guangxi Province China; 2 Affiliated Liutie Central Hospital of Guangxi Medical University, Liunan Distract, Liuzhou, Guangxi China; 3 Affiliated Primary School Liugong Middle School, Liunan Distract, Liuzhou, Guangxi China

**Keywords:** Stroke, Gastrointestinal haemorrhage, Antiplatelet drugs, Unsupervised machinery learning

## Abstract

**Background:**

For ischaemic stroke patients with gastrointestinal haemorrhage, stopping antiplatelet drugs or reducing the dose of antiplatelet drugs was a conventional clinical therapy method. But not a study to prove which way was better. And the machinery learning methods could help to obtain which way more suit for some patients.

**Methods:**

Data from consecutive ischaemic stroke patients with gastrointestinal haemorrhage were prospectively collected. The outcome was a recurrent stroke rate, haemorrhage events, mortality and favourable functional outcome (FFO). We analysed the data using conventional logistic regression methods and a supervised machine learning model. We used unsupervised machine learning to group and analyse data characters.

**Results:**

The patients of stopping antiplatelet drugs had a lower rate of bleeding events (*p* = 0.125), mortality (*p* = 0.008), rate of recurrence of stroke (*p* = 0.161) and distribution of severe patients (mRS 3–6) (*p* = 0.056). For Logistic regression, stopping antiplatelet drugs (OR = 2.826, *p* = 0.030) was related to lower mortality. The stopping antiplatelet drugs in the supervised machine learning model related to mortality (AUC = 0.95) and FFO (AUC = 0.82). For group by unsupervised machine learning, the patients of better prognosis had more male (*p* < 0.001), younger (*p* < 0.001), had lower NIHSS score (*p* < 0.001); and had a higher value of serum lipid level (*p* < 0.001).

**Conclusions:**

For ischemic stroke patients with gastrointestinal haemorrhage, stopping antiplatelet drugs had a better prognosis. Patients who were younger, male, with lesser NIHSS scores at admission, with the fewest history of a medical, higher value of diastolic blood pressure, platelet, blood lipid and lower INR could have a better prognosis.

**Supplementary Information:**

The online version contains supplementary material available at 10.1186/s12883-023-03422-0.

## Introduction

Antiplatelet drugs were a guideline-recommended secondary prevention method for ischemic stroke patients [1]. It also had many sides effect, such as gastrointestinal toxicity and haemorrhage events [2]. Gastrointestinal haemorrhage was a common complication for stroke patients with antiplatelet drugs [3].

When stroke patients occurred gastrointestinal haemorrhage, the antiplatelet drugs are discontinued temporarily, or reduced doses of drugs prevent the progression of bleeding. A study suggested that stopping antiplatelet medicines for more than ten days could bring a higher risk of ischemic stroke, for the remnant drugs in the blood could not effectively inhibit platelet circulation [4]. The second method, reducing the dose of antiplatelet medicines, may prevent an ischemic stroke attack. Still, the lower dose of drugs did not reduce the risk of haemorrhage events for patients [5]. Therefore, there was no best method for ischemic stroke patients with gastrointestinal haemorrhage, especially since there had no definite study evidence to compare the effect of the two ways.

As mentioned above, many studies had different conclusions for the same research question. Machinery learning methods were better for dealing with these conditions [6]. Factor analysis and reduction of dimensionality could effectively deal with complicated confounders and collinearity of a great deal of character [7]. Unsupervised machine learning could analyse and classify the prospective data [8]. And the analysis and classification could be more suit for clinical patients in hospitals [9].

In our study, we observed the relationship between discontinued temporarily or reduced antiplatelet doses of drugs with stroke patients with gastrointestinal haemorrhage. Then we deal with data with a supervised machine learning model. At last, we used unsupervised machine learning methods to group and explore the character of patients.

## Methods

### Patients

The study is a prospective observational cohort design. We consecutively recruited acute ischemic stroke patients who occurred gastrointestinal haemorrhage within 30 days after onset. All patients were from the Neurology Department and Rehabilitation Department of Affiliated Liutie Central Hospital of Guangxi Medical University. The patients were enrolled from January 1, 2017, to March 30, 2022, and followed up until June 30, 2022. All patients’ diagnoses met the WHO stroke diagnostic criteria, which included clinical character and neurological image examination. The gastrointestinal haemorrhage had hematemesis, hematochezia and tarry stool. The gastrointestinal haemorrhage was proven through clinical features and laboratory examination by experienced neurologists or gastroenterologists.

The patients who stopped antiplatelet drugs after gastrointestinal haemorrhage were assigned to stopped group. And those who reduced the dose of antiplatelet medicines were assigned to reduced group. The different therapeutic schedule of patients was for their various conditions or evaluated by their attending doctor. The time range of stopping antiplatelet drugs was about 15 days for patients. The dose of aspirin or clopidogrel in reduced group was 25 mg twice or 50 mg once daily. We are calculated the sample size At the α = 5%, 1- β = 0.80 level by SAS based on a previous article [3]. Considering a 10% loss to follow-up, we initially recruited more than 200 patients.

The inclusion criteria were as follows: (1) aged 18 years or older; (2) having received an antiplatelet drug and other conventional therapy after admission; (3) gastrointestinal haemorrhage occurred within 30 days after onset.

The exclusion criteria were as follows: (1) patients with a recent history of peptic ulcer or gastrointestinal bleeding before onset; (2) taking anticoagulant drugs; (3) patients with intracerebral haemorrhage, subarachnoid haemorrhage or severe systemic disease. (4) patients withdraw study or cannot provide outcome events.

### Data collected and outcome

Baseline data were collected from electronic clinical records and structured questionnaires after admission. The detail of the data was in the baseline character table (Table 1). Due to the narrow inclusion criteria, the type of patients was lacunar infarction (*n* = 71, 26.49%) and large-artery atherosclerosis ischemic stroke (*n* = 197,73.51%), the study did not include atrial fibrillation or other patients cardioembolic Stroke patients.

**Table 1:** Baseline characteristic

variables	Stopped group (*N* = 176)	Reduced group (*N* = 92)	P*
Age, years	67.45(13.15)	66.13(14.16)	0.447
Female, %	71(40.3)	37(40.2)	0.545
Admission NIHSS score	11.55(7.01)	12.78(7.60)	0.184
Endovascular Treatment, %	61(34.7)	26(28.3)	0.178
Taking aspirin, %	96(54.5)	54(59.7)	0.483
Statins In Hospital, %	168(95.5)	88(95.7)	0.949
Length of stay, days, mean (SD)	14.34 (2.21)	18.23 (1.92)	0.067
Systolic Blood Pressure, mmHg	146.96(25.06)	144.15(26.60)	0.395
Diastolic Blood Pressure, mmHg	83.40(16.01)	83.11(17.67)	0.859
History of Smoking, %	67(38.1)	35(38.0)	0.402
History of Drinking, %	34(19.3)	14(15.2)	0.387
History of Stroke, %	17(9.7)	7(7.6)	0.377
History of Hypertension, %	79(44.9)	49(53.3)	0.120
History of Diabetes Mellitus, %	29(16.5)	13(14.1)	0.377
History of CHD, %	20(11.4)	13(14.1)	0.628
History of Antiplatelet Drug, %	20 (11.4)	10(10.9)	0.539
History of Statins, %	14(8.0)	8(8.7)	0.914
History of Antihypertensive, %	58(33.0)	40(43.5)	**0.021**
History of Hypoglycaemic, %	20(11.4)	12(13.0)	0.526
Platelet, mmol/l	180.30(60.22)	174.80(56.80)	0.470
INR	0.98(0.09)	1.01(0.14)	0.101
ALT, mmol/l	22.82(14.02)	23.07(15.37)	0.895
Creatinine, mmol/l	79.22(26.47)	78.59(22.65)	0.846
Glucose, mmol/l	7.99(2.68)	8.18(3.56)	0.614
Triglyceride, mmol/l	1.68(1.50)	1.69(1.18)	0.960
Total cholesterol, mmol/l	4.36(1.00)	4.34(1.11)	0.907
HDL-C, mmol/l	1.28(0.40)	1.24(0.33)	0.422
LDL-C, mmol/l	2.58(0.84)	2.62(0.92)	0.733

P* was calculated by ANOVA, Chi-square test as appropriate. *CHD* Coronary Heart Disease, *INR* international normalized ratio, *ALT* glutamic-pyruvic transaminase, *HDL-C* high-density lipoprotein cholesterol, *LDL-C* low-density lipoprotein

The primary outcome was a recurrent stroke rate within 90 days after onset. All recurrence patience had a conventional therapy in hospital base on their condition. The second outcome included haemorrhage events, mortality within 90 days after onset and favourable functional outcome at 90 days after onset. The favourable functional outcome (FFO) was defined as mRs <  = 2 at 90 days after admission. The unfavourable functional outcome (UFO) was defined as mRs > 2 at 90 days after admission. The haemorrhage events included intracerebral haemorrhage and gastrointestinal haemorrhage. The score of clinical scale and outcome events were determined by experienced neurologists blind to the patients’ group.

### Statistical analysis and machine learning

#### Baseline data

We process the data through SPSS 23.0 for Windows (conventional statistical analysis) and Python 3.80 (machine learning). We used a t-test to analyse continuous variables following a normal distribution. For abnormally distributed continuous variables, we used a non-parametric test. A chi-square test was conducted for categorical data and ranked data.

#### Outcome events

We compared the events of group differences using the chi-square test. We analysed the relationship between risk factors such as difference group and outcome events using conventionally univariable and multivariable logistic regression methods. We screen the significant risk factors by univariable logistic regression methods (*P* <  = 0.0.05 or factor with clinical significance). These factors would bring into multivariable logistic regression methods and supervised machine learning.

#### Supervised machine learning

We train all data (including outcomes events) by logistic regression methods, support vector machine (SVM) and decision trees methods, voting classifier and random forest. We compare model performance through precision and area under the curve (AUC) plot of each model. Then we evaluated the importance of variables in the model.

#### Unsupervised machine learning

We analyzed all baseline data by cluster analysis. We cluster baseline by K-means methods and Hierarchical Clustering. Then we choose the best number of groups by the two methods (the detail of Unsupervised machine learning in Supplemental Material).

We compare outcome events by the above difference group. Most outcome events between groups had a significant difference and were the best choice for grouping. Then we analyzed the different characteristics of factors in the group.

## Results

### Baseline character

Initially, the study recruited 300 patients. Among them, eight patients were no longer suited for inclusion criteria, 15 were lost to follow-up, and nine withdrew from the study. Finally, we acquired the data from 268 patients (176 in stopped group and 92 in reduced group). Among the 268 patients (mean age: 67.00 ± 13.49 years), 113 (40.3%) were female. Stopped group had a lower rate of patients with a history of antihypertensive drugs than reduced group. The other baseline data between the two groups had not shown a significant difference (Table 1).

### Primary outcome events

**Fig. 1:**
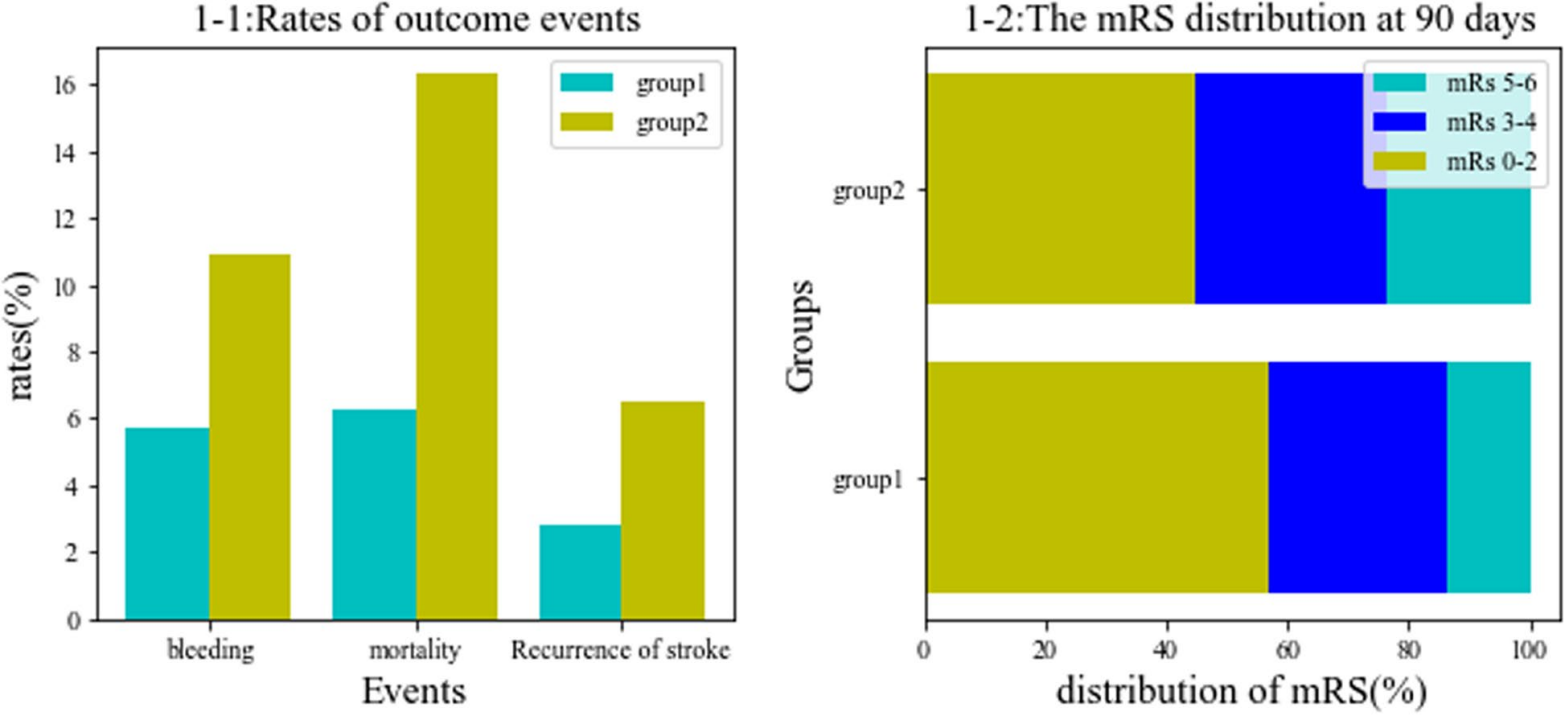
Difference rates of outcome between stopped group and reduced group

As shown in Fig. 1a, to compare with reduced group (recurrence patients = 6, ischemic stroke = 5, hemorrhagic stroke = 1), the patients of stopped group (recurrence patients = 5, ischemic stroke = 5) had lower rate of recurrence of stroke (*p* = 0.161), but the difference had not a statistic significant.

In the univariate logistic regression analysis, we could not acquire the relationship between risk factors and the recurrence of stroke events or bleeding events.

### Second outcome events

As shown in Fig. 1a, to compare with reduced group, the patients of stopped group had lower mortality (*p* = 0.008) and a lower rate of bleeding events (*p* = 0.125), but the last results had not a statistic significant. As shown in Fig. 1b, to compare with reduced group, the patients of stopped group had a lower rate distribution of severe patients (mRS 3–6) at 90 days (*p* = 0.056) but the results had not a statistic significant.

In the univariate logistic regression analysis, when we analysed risk factors of mortality, we found that older age (OR = 1.070, *p* = 0.001), female (OR = 2.609, *p* = 0.024), and higher NIHSS scores at admission (OR = 2.812, *p* < 0.001), patients in reduced group (OR = 2.922, *p* = 0.011) were related to the higher mortality within 90 days after admission. Smoking (OR = 0.287, *p* = 0.017) and using statin (OR = 0.390, *p* = 0.023) negatively correlated with higher mortality within 90 days after admission. When we analysed the risk factor of UFO, we found that older age (OR = 1.041, *p* < 0.001), female (OR = 1.735, *p* = 0.028), patients in reduced group(OR = 1.220, *p* < 0.001) and higher NIHSS scores at admission (OR = 1.892, *p* < 0.001) were related to the UFO at 90 days after admission. Smoking (OR = 0.666, *p* = 0.044), using statin (OR = 0.465, p = 0.002), higher value of triglyceride (TG) (OR = 0.287, *p* = 0.017) and higher value of glutamic-pyruvic transaminase (ALT) (OR = 0.977, *p* = 0.015) at admission were negative correlation with the UFO at 90 days after admission.

By the multivariable logistic regression analysis, we found that the older age (OR = 1.060, *p* = 0.009), higher NIHSS scores at admission (OR = 2.179, *p* = 0.008) and patients in reduced group (OR = 2.826, *p* = 0.030) were still related to the higher mortality within 90 days after admission. The older age (OR = 1.028, *p* = 0.021), patients in reduced group (OR = 0.621, *p* = 0.044) and higher NIHSS scores at admission (OR = 1.090, p < 0.001) were still related to the unfavourable functional outcome at 90 days after admission.

### Supervised machine learning

We explored the relationship between risk factors and mortality and FFO with a machine learning model. The support vector machine model, including six factors, had the best performance for mortality (the accuracy score = 0.952), and the support vector machine model, including three factors, had the best performance for FFO (the accuracy score = 0.652).

**Fig. 2:**
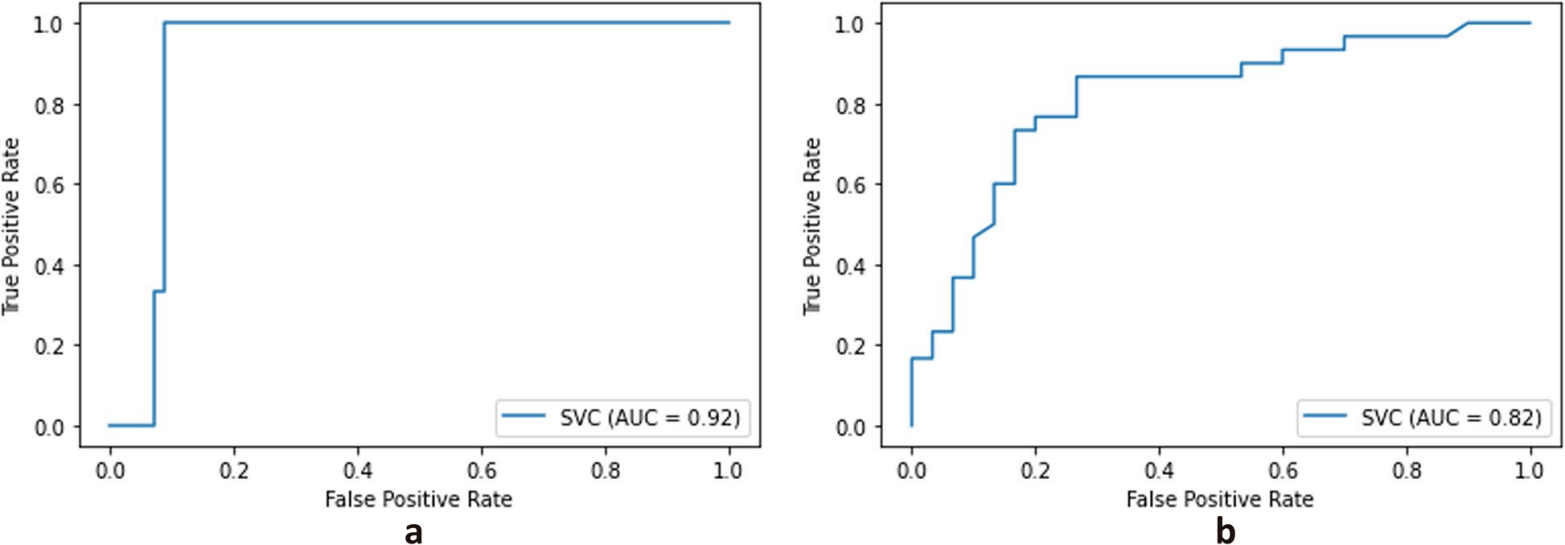
**a** The AUC plot of the support vector machine model for mortality; **b **The AUC plot of the support vector machine model for FFO. AUC area under the curve; FFO favourable functional outcome

The final support vector machine model for mortality showed that its AUC was 0.92, and prediction accuracy was 0.95 (Fig. 2a). The final support vector machine model for FFO showed that its AUC was 0.820, and prediction accuracy was 0.78 (Fig. 2b). The coefficient of the model is displayed in Table 2.

**Table 2:** The coefficient of support vector machine model

Risk factor	Mortality Within 90 days	FFO at 90 days
	**coefficient**	**coefficient**
Older	0.033	-0.018
Higher NIHSS at admission	1.776	-0.110
stopping antiplatelet drugs	-2.040	0.164
Female	2.403	
Using statins	-1.576	
Smoking	-1.755	

The coefficient was calculated by support vector machine model

### Unsupervised machine learning

**Fig. 3:**
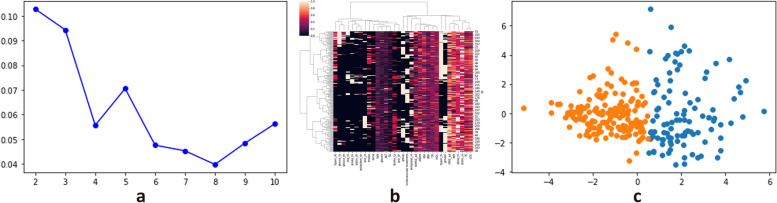
**a** The line chart of Silhouette score for k-means methods; **b** The hot map for hierarchical clustering methods; **c ** The two-dimension scatter plot of 2 groups by k-means

As shown in Fig. 3a, the line chart of Silhouette score showed that the 2 group was the best choice. The hot map showed that the 2 group was better for the Hierarchical Clustering model (Fig. 3b). The two-dimension scatter plot showed that the two groups by k-means had more precise discrimination (Fig. 3c).

**Fig. 4:**
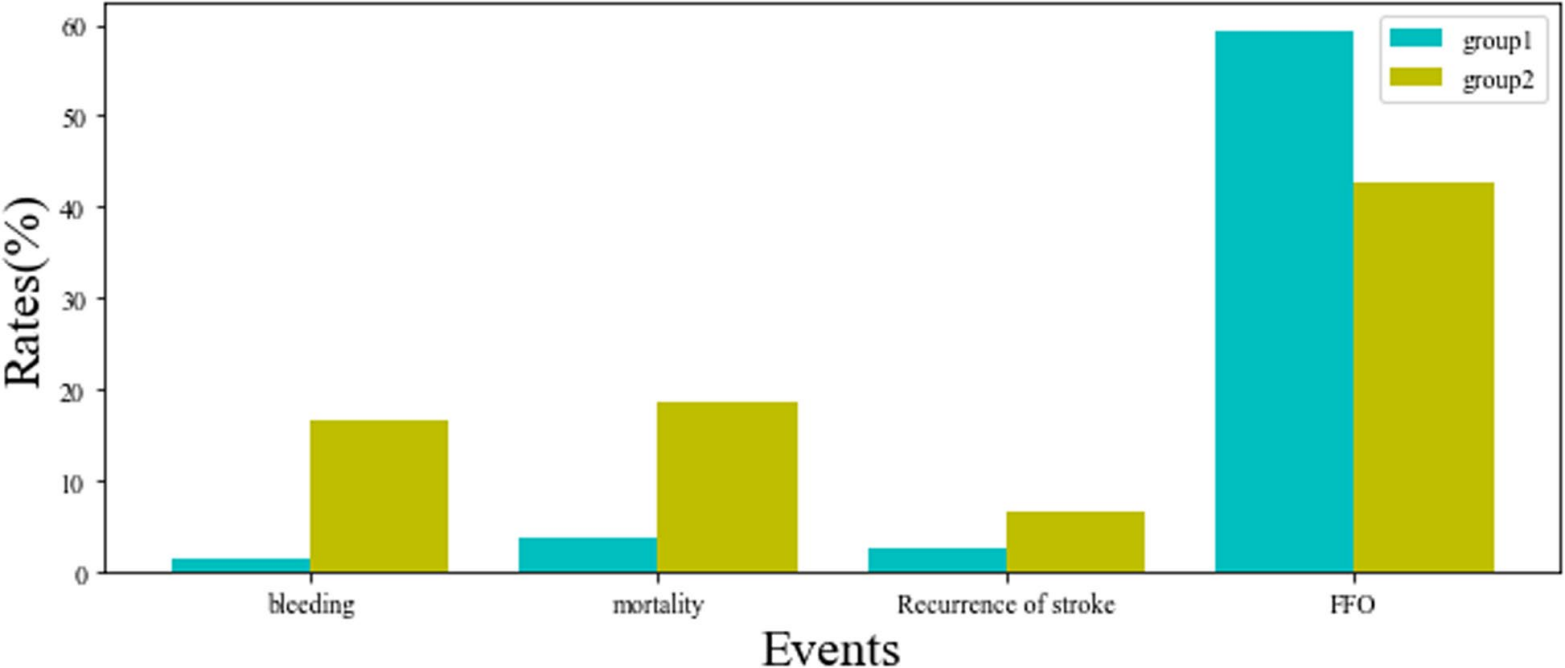
Difference rates of outcome between km2-1 group and km2-2 group

We grouped data into km2-1group and km2-2 group by K-means methods. We grouped data into hc2-1 group and hc2-2 group by the hierarchical clustering methods. The outcome event rate had not shown a significant difference between hc2-1 group and hc2-2 group. The km2-1 group had a lower rate of bleeding events (*p* < 0.001), lower mortality (*p* < 0.001), a lower rate of recurrence of stroke (*p* = 0.107) and a higher rate of FFO (*P* = 0.007) than km2-2 group (Fig. 4). Therefore, the 2 group by K-means method was the best grouping method.

To compare with km2-2 group, the patients of the km2-1 group had lesser female patients, lesser older patients, higher diastolic blood pressure at admission, lower NIHSS score at admission, higher rate of statin used, lower rate of disease history, higher value of platelet at admission and higher value of blood lipid at admission (Table 3).

**Table 3:** Difference character between Km2-1 group And Km2-2 group

Variables	km2-1 group (*N* = 160)	km2-2 group (*N* = 108)	P*
Age, years	62.78(13.19)	73.26(11.38)	** < 0.001**
Female, %	43(26.9)	65(60.2)	** < 0.001**
Admission NIHSS score	10.22(6.50)	14.56(7.50)	** < 0.001**
Statins In Hospital, %	158(98.7)	98(90.7)	** < 0.001**
Diastolic Blood Pressure, mmHg	85.91(16.88)	79.58(15.42)	**0.002**
History of Smoking, %	80(50.0)	22(20.4)	** < 0.001**
History of Drinking, %	43(26.9)	5(4.6)	** < 0.001**
History of Stroke, %	8(5.0)	16(14.8)	**0.006**
History of Hypertension, %	66(41.3)	62(57.4)	**0.009**
History of Diabetes Mellitus, %	17(10.6)	25(23.1)	**0.006**
History of CHD, %	7(4.4)	26(24.1)	** < 0.001**
History of Antiplatelet Drug, %	8 (5.0)	22(20.4)	** < 0.001**
History of Antihypertensive, %	40(25.0)	58(53.7)	** < 0.001**
History of Hypoglycaemic, %	10(6.2)	22(20.4)	**0.003**
Platelet, mmol/l	185.21(54.83)	168.35(63.67)	**0.022**
INR	0.96(0.08)	1.05(0.13)	** < 0.001**
ALT, mmol/l	24.56(15.84)	20.44(11.80)	**0.022**
Triglyceride, mmol/l	1.97(1.67)	1.25(0.63)	** < 0.001**
Total cholesterol, mmol/l	4.77(0.95)	3.74(0.84)	** < 0.001**
LDL-C, mmol/l	2.93(0.80)	2.08(0.70)	** < 0.001**

P* was calculated by ANOVA, Chi-square test as appropriate. *CHD* Coronary Heart Disease, *INR* international normalized ratio, *ALT* glutamic-pyruvic transaminase, *LDL-C* low-density lipoprotein

## Discussion

Our study found that for ischemic stroke patients with a gastrointestinal haemorrhage, stopping antiplatelet drugs had a lower rate of bleeding events, mortality, rate of recurrence of stroke and a higher rate of FFO than reducing the dose of antiplatelet medicines. Stopping antiplatelet drugs is related to a higher rate of FFO and lower mortality. The machine learning model also showed that stopping antiplatelet drugs affects patients' mortality and FFO. The group by unsupervised machine learning methods suggested that many risk factors significantly differed for different outcome events groups.

The stopping antiplatelet drugs had a lower rate of bleeding events and stroke recurrence rate for patients. However, the difference was not statistically significant for the fewer events. Our results suggested that stopping antiplatelet drug had a better effect and safety than reducing the dose of antiplatelet drug. One study showed that discontinuous antiplatelet drugs did not affect patient outcomes [4]. Another study suggested that lower doses of antiplatelet drugs did not reduce the risk of bleeding events for ischemic stroke patients [5]. These studies all were consistent with our research. The difference type of antiplatelet drug had not a significant difference in two group and the type had not relate to all outcome events.

For mortality and FFO outcomes, it was stopping antiplatelet drugs related to better outcomes through univariable and multivariable logistic regression methods. The coefficient of the machine learning model suggested that stopping antiplatelet drugs affected predicting mortality and FFO for ischemic stroke patients with gastrointestinal haemorrhage. These results proved the significant relationship between stopping antiplatelet drugs and lower mortality and FFO. Some studies also had similar results for ischemic stroke or coronary artery disease patients [10].

When we analysis the difference in the km2-1 and km2-2 group. The km 2–1 group had a better prognosis. We found that younger or lower NIHSS scores had a better outcome, consistent with other studies and guides [1, 11, 12]. Meanwhile, the patients using statin had a better outcome, consistent with many studies, including our research [13–15]. The km2-1 group had fewer patients who had strokes, and other medical histories also were reasonable for the patients with medical history had a worse condition. Therefore, the km2-1 group had fewer patients with a history of using drugs for less medical history. The female had a worse outcome, consistent with some recent studies and our study [15, 16]. The results also could explain that the km2-1 group had more patients smoking and drinking. So, we should realize that female patient had a worse prognosis though they had lower morbidity of ischemic stroke. The km2-1 group patients had higher diastolic blood pressure at admission, which was also unusual. The patients with higher diastolic blood pressure may have a better condition than those with lower blood pressure because the mean value of diastolic blood pressure in the km2-2 group is less than normal values (80 mmHg).

To analysis the difference in laboratory data between the km2-1 group and the km2-2 group, we found that the patients of the km2-1 group with a higher value of platelet and a lower value of INR at admission were reasonable for a better outcome. Patients with bleeding events obviously could benefit from better coagulation function. The patients of the km2-1 group with a higher value of glutamic-pyruvic transaminase at admission had a complex reason. The value in the two groups all was in normal values. Therefore, the lower value of glutamic-pyruvic transaminase may suggest that patients had poor conditions such as malnutrition. It also could explain that patients of the km2-1 group had a higher value of serum lipid level (TG, TC, LDL). The lipid contradiction also might partly explain the condition [17]. Though a higher serum lipid level was a risk factor for an ischemic stroke attack, the ischemic stroke patients with a higher serum lipid level had a better outcome, such as lower mortality and rate of haemorrhage events [18]. However, the conclusion needs further study to prove. We may have a caution for lower serum lipid levels for ischemic stroke patients with gastrointestinal haemorrhage.

Our study has several limitations. First, our machine-learning model did not verify by external data. Therefore, the extensionality of the model needs to be further proved. But our study focused on the relationship between factors and outcomes. The extensionality of the model did not affect the statistical power of the relationship. Second, the results of unsupervised machine learning also did not verify by external data. However, the results were confirmed by the outcome events of our data. To further prove these results with more data was reasonable.

In conclusion, for ischemic stroke patients with gastrointestinal haemorrhage, patients stopping antiplatelet drugs had a lower rate of mortality and a higher rate of FFO than patients of reducing the dose of antiplatelet medicines. For grouping by unsupervised machine learning methods, younger, male, with lesser NIHSS scores at admission, with the fewest history of a medical and slightly higher value of diastolic blood pressure, with higher value of platelet, lower value of INR and higher value of blood lipid could have a better prognosis.

### Supplementary Information

**Additional file 1.** 

## Data Availability

The datasets used and analysed during the current study are available from the corresponding author upon reasonable request.
