# Supplementary material for: The machine learning methods to analyze the using strategy of antiplatelet drugs in ischaemic stroke patients with gastrointestinal haemorrhage
Source: BMC Neurol. 2023 Oct 13;23:369. doi: 10.1186/s12883-023-03422-0 (PMC10571309; doi:10.1186/s12883-023-03422-0)
Supplement: Supplementary file 1 — Additional file 1. [file 12883_2023_3422_MOESM1_ESM.docx]

**Unsupervised machine learning**

we analyzed all baseline data by cluster analysis. We cluster baseline by two models: K-means methods (K-Means module, sklearn library) and Hierarchical Clustering (AgglomerativeClustering module, sklearn library). First, we used principal component analysis (PCA), reducing the dimensions of the baseline data to two-dimension (PCA module, sklearn library). Then we could draw a scatter plot for the two-dimension data. We draw a line chart by Silhouette score. Through the line chart, we choose the best number of groups in the K-means method. Then we draw a scatter plot by the number of groups. We could further evaluate whether the number of groups in the K-means methods was reasonable by the scatter plot. We draw a hot map by the character of baseline data. We choose the best number of groups in Hierarchical Clustering through the hot map.
